# Acute neural effects of the mood stabiliser lamotrigine on emotional processing in healthy volunteers: a randomised control trial

**DOI:** 10.1038/s41398-024-02944-6

**Published:** 2024-05-27

**Authors:** Marieke A. G. Martens, Tarek Zghoul, Evelyn Watson, Sebastian W. Rieger, Liliana P. Capitão, Catherine J. Harmer

**Affiliations:** 1 Department of Psychiatry, University of Oxford, Oxford, UK; 2 Oxford Health NHS Foundation Trust, Oxford, UK; 3 Wellcome Centre for Integrative Neuroimaging, University of Oxford, Oxford, UK; 4 Institute of Sport Exercise and Health, Faculty of Medical Sciences, University College London, London, UK; 5 Institute of Cognitive Neuroscience, Faculty of Brain Sciences, University College London, London, UK; 6 Psychology Research Centre (CIPsi), School of Psychology, University of Minho, Braga, Portugal

**Keywords:** Bipolar disorder, Human behaviour

## Abstract

Lamotrigine is an effective mood stabiliser, largely used for the management and prevention of depression in bipolar disorder. The neuropsychological mechanisms by which lamotrigine acts to relieve symptoms as well as its neural effects on emotional processing remain unclear. The primary objective of this current study was to investigate the impact of an acute dose of lamotrigine on the neural response to a well-characterised fMRI task probing implicit emotional processing relevant to negative bias. 31 healthy participants were administered either a single dose of lamotrigine (300 mg, *n* = 14) or placebo (*n* = 17) in a randomized, double-blind design. Inside the 3 T MRI scanner, participants completed a covert emotional faces gender discrimination task. Brain activations showing significant group differences were identified using voxel-wise general linear model (GLM) nonparametric permutation testing, with threshold free cluster enhancement (TFCE) and a family wise error (FWE)-corrected cluster significance threshold of *p* < 0.05. Participants receiving lamotrigine were more accurate at identifying the gender of fearful (but not happy or angry) faces. A network of regions associated with emotional processing, including amygdala, insula, and the anterior cingulate cortex (ACC), was significantly less activated in the lamotrigine group compared to the placebo group across emotional facial expressions. A single dose of lamotrigine reduced activation in limbic areas in response to faces with both positive and negative expressions, suggesting a valence-independent effect. However, at a behavioural level lamotrigine appeared to reduce the distracting effect of fear on face discrimination. Such effects may be relevant to the mood stabilisation effects of lamotrigine.

## Introduction

Bipolar disorder (BD) is among the top causes of worldwide disability, with significant associated morbidity and mortality [1, 2]. It usually presents during adolescence or early adulthood and affects 3% of the world’s population [3]. First line treatment for BD includes monotherapy or adjunctive treatment with mood stabilisers such as lithium and various anticonvulsants and antipsychotics [4]. However, treating BD is often complicated by initial misdiagnosis as unipolar depression and subsequent treatment with conventional antidepressants, which carry a high risk of mood destabilisation in this group [5]. The mood stabiliser lamotrigine is however generally successful in BD therapy at treating the depressive phase without inducing mania [6]. Initially synthesized in the 1980s as an antiepileptic drug, lamotrigine is also approved by the US Food and Drug Administration(FDA) and European Medicines Agency (EMA) for the long-term maintenance treatment of BD [3, 7].

Despite its widespread use, lamotrigine’s mode of action as a mood stabiliser has yet to be fully elucidated. One hypothesis is that lamotrigine exerts its mood stabilising effects in a way that is similar to its antiepileptic properties [8], by inhibiting voltage-gated sodium channels and reducing downstream glutamate release. Whole-cell patch clamp recordings from granule cells in the dentate gyrus have shown that lamotrigine inhibits glutamate release in preclinical models [9]. Functional magnetic resonance imaging (fMRI) studies in humans have also shown that lamotrigine pre-treatment attenuates the neural effects of the glutamate release promoter ketamine [10, 11]. Further, studies using the forced swim test animal model of depression have shown that pre-treatment with the voltage-gated sodium channel opener veratrine reverses lamotrigine’s antidepressant effects [12]

The neuropsychological mechanisms that underpin lamotrigine’s mood stabilising effects are however less clear. For example, it is not known whether lamotrigine has effects on emotional information processing that are comparable to those of conventional antidepressant drugs, like selective serotonin reuptake inhibitors (SSRIs). As for unipolar depression, there is evidence that patients with bipolar disorder have altered abnormal emotional information processing which may elicit and maintain a depressive episode [13–15]. According to the cognitive neuropsychological model of antidepressant drug action, antidepressants target emotional processing rather than mood directly [16–18]. They do this via an ability to (sub-) acutely shift the processing of emotional information away from a preference for negative relative to positive input [16]. This induced bias in favour of positive emotional material is thought to counterbalance negatively biased emotional information processing associated with depression. On the neural level this is reflected in antidepressant drug effects which have been reported to decrease limbic and anterior cingulate cortex (ACC) activity in response to negative versus positive information, as well as increasing engagement of prefrontal cortex (PFC) areas such as the dorsolateral PFC (dlPFC) [19, 20]. These valence-dependent effects have been observed both in patients with depression as well as in healthy volunteers, suggesting that they are an inherent effect of antidepressant treatment and not simply due to subtle improvements in psychopathology [20]. Importantly, these changes in emotional processing are typically observed long before improvements in mood symptoms normally become apparent in patients with depression and these effects are associated with later clinical response, suggesting that they represent a critical pathway through which antidepressants exert their effects [19, 21–25].

The current study investigated whether lamotrigine affects neural response to emotional stimuli in healthy volunteers using a well validated fMRI paradigm known to reliably elicit amygdala activity. Healthy volunteers were selected in this study to investigate the direct effects of lamotrigine unconfounded by disorder-related factors such as symptom severity and previous antidepressant exposure, as well as potential symptom improvement following lamotrigine administration which would also be expected to affect emotional processing bias. We hypothesised that lamotrigine would attenuate amygdala response to negative emotional stimuli and/ or increase the response to positive emotional stimuli when compared to placebo, similar to the profile seen with conventional antidepressant drugs.

## Materials and methods

### Participants

**Fig. 1:**
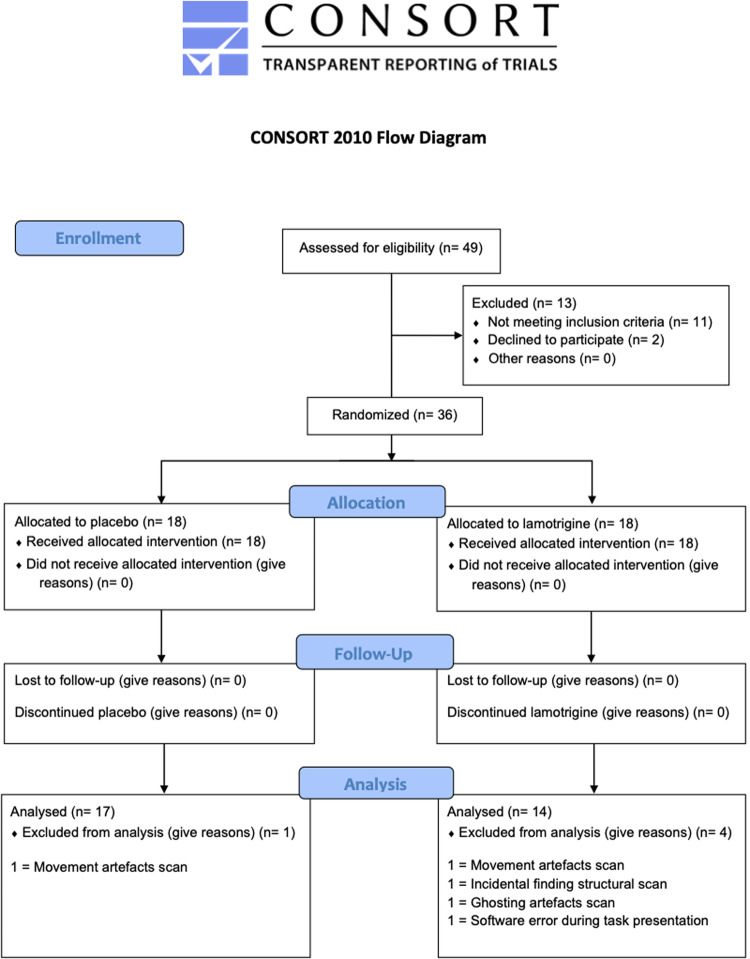
CONSORT diagram to show flow of participants through the study.

Ethical approval was granted by the University of Oxford Central University Research Ethics Committee (CUREC R49749/RE003) and the protocol was pre-registered with clinicaltrials.gov (NCT04396938). Thirty-six healthy adult volunteers (24 men, 12 women, mean age 24.11 ± 4.43 years, range 18 to 32 years) recruited from the Oxfordshire community took part in this study. The flow of participants is outlined in Fig. 1. Participants were recruited through advertising and screened for eligibility. Details of inclusion and exclusion criteria (pre-established) are included in Supplementary Material. In brief, recruited participants were fluent in English, healthy, not pregnant or breastfeeding and not taking any psychoactive medication. All participants gave written informed consent. No changes to methods occurred after start of the study. A formal sample size calculation was precluded, because no prior study had determined the acute effect of lamotrigine on brain activity in healthy volunteers. Sample size was therefore modelled on previous successful studies using a similar design. Acute citalopram was found to reduce amygdala activation with an effect size of 1.19 (anatomically defined region of interest analysis) [26]. Informed by these data, an a priori sample size calculation for the current between-subjects design yielded *n* = 13 as the minimum sample size required to detect a reduction in amygdala fMRI signal of this magnitude (difference between two independent means: two tailed, alpha = 0.05, effect size = 1.19, power = 0.8).

### Procedures and measures

The study had a between-subject, double-blind, placebo-controlled design. Eligible participants were randomly allocated to one of two treatment conditions: a single oral dose of lamotrigine (300 mg) or placebo (lactose capsule). Randomisation was blocked in design (block size = 4), stratified for sex and undertaken with an online tool (sealed envelope). A researcher not involved in the study was responsible for randomisation and encapsulation. Group allocation was concealed from participants, investigators and assessors using sequential numbered containers identical in packaging and appearance.

**Fig. 2:**
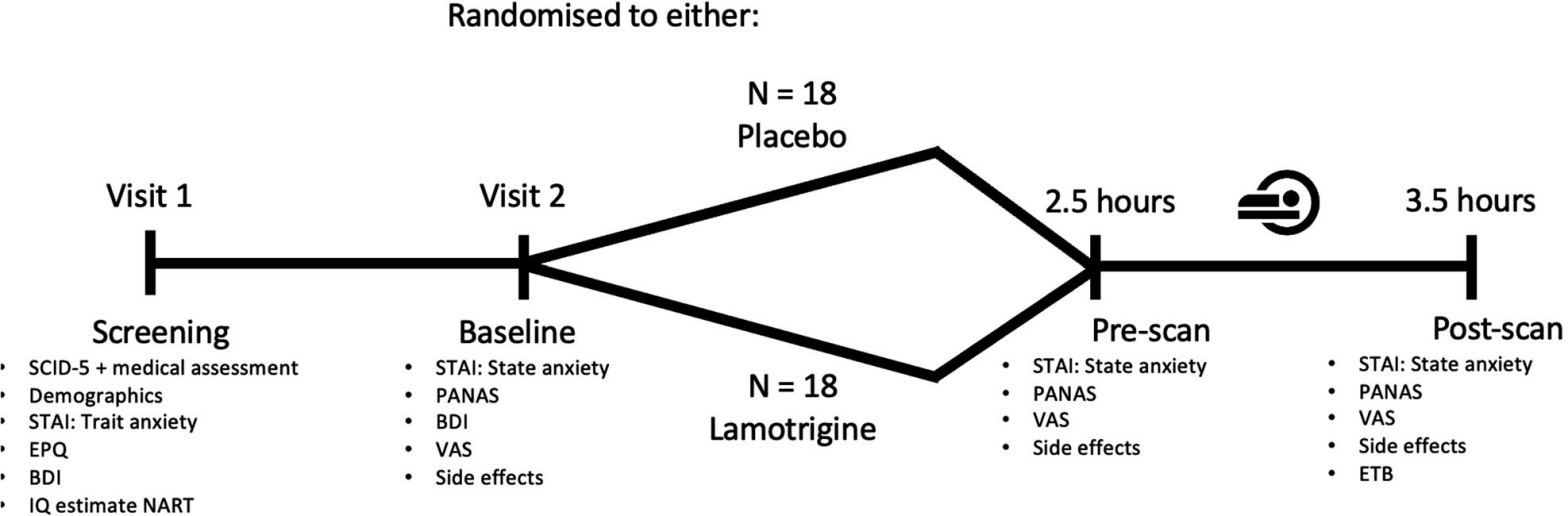
Study flow diagram.

The study flow diagram is presented in Fig. 2. At the start of the research visit, female participants completed a pregnancy test. Participants were assessed for the following baseline measurements: State-Trait Anxiety Inventory (STAI), Positive and Negative Affect Schedule (PANAS), Eysenck Personality Questionnaire (EPQ), Beck Depression Inventory (BDI) and visual analogue scales for alertness, calmness, and satisfaction (VAS – [27]) (all secondary outcomes). Following completion of baseline questionnaires, lamotrigine or placebo was administered. Participants spent 2 h in a quiet room after which they completed pre-scan questionnaires and mood scales (STAI, PANAS, VAS, secondary outcomes) when the plasma concentration of lamotrigine would be expected to be at its peak [28]. A 60-minute MRI scan was then completed, including a structural scan, visual checkerboard stimulation paradigm (secondary outcome), the covert emotional faces gender discrimination task (primary outcome) and a resting state scan (reported elsewhere). Following the scan, participants completed the Emotional Task Battery (ETB) (reported in [29]). After the fMRI (post-scan), participants again completed the STAI, VAS, and PANAS as endpoint measurements of subjective mood and experience. Side effects (secondary outcome) were quantified at each of three time points as well (baseline, pre-scan, post-scan) using a non-validated semi-qualitative self-report rating scale (absent=0, mild=1, moderate=2, severe=3) for the following symptoms: nausea, dry mouth, agitation, aggression, headache, drowsiness, dizziness, tremor, back/joint pain, vision impairment, and rash.

### Behavioural data analysis

Statistical analysis was performed using IBM SPSS Statistics version 26 for Windows. Questionnaires and scales measuring mood changes throughout the experiment, side-effects, response accuracy, and response time were analysed using mixed repeated-measures ANOVA. Treatment group (two levels: lamotrigine and placebo) was the between-subjects factor and either time point (three levels: baseline, pre-scan, post-scan) or subjective experience (fear, happy, anger or nausea, dry mouth, agitation, aggression, headache, drowsiness, dizziness, tremor, back/joint pain, vision, rash) was the within-subjects factor. Huynh-Feldt correction was applied where data failed Mauchly’s Test of Sphericity. Mean and SD are reported for behavioural measures. A p value < 0.05 was used to denote statistical significance. Adjustments for multiple testing were not made as potential group differences on most of these behavioural and subjective measures (like anxiety and side effects) would complicate interpreting the neural results. Findings are therefore presented uncorrected, with potential confounds included in the analysis as regressors / covariates of no interest.

### fMRI

#### fMRI data acquisition

Scanning was performed at the Oxford Centre for Human Brain Activity (OHBA), University of Oxford, using a 3-Tesla Siemens Prisma scanner with a 32-channel head-coil. See supplementary information for the neuroimaging protocol.

#### fMRI task designs

The faces task (also called gender discrimination task, described in full and previously used in similar studies) is a covert task designed to probe emotional processing and has proved sensitive to the acute effects of antidepressants on neural processing [30]. The task was a block design presenting colour photographs of faces expressing three emotions (fear, happy, anger) taken from the NimStim database [31]. For more details, please see supplementary information. Participants were asked to respond by indicating the gender (male or female) of each face as quickly and accurately as possible via button press. Reaction time (total time between face stimuli presentation and gender classification response) and accuracy (number of faces correctly identified as male/female divided by total number of faces) were measured and used as a measure of task engagement. Following the faces task, a checkerboard visual paradigm was presented. This assessed the effect of lamotrigine on the blood oxygen level-dependent (BOLD) signal in the primary visual cortex, to control for a possible confounding effect of global drug-related modulation of BOLD signal. For more details, please see supplementary information.

#### fMRI data analysis

Data were analysed using FSL (FMRIB Software Library v6.0) tools (https://fsl.fmrib.ox.ac.uk/fsl).

fMRI data were pre-processed and analysed using FEAT (FMRI Expert Analysis Tool). For further information on the pre-processing please refer to the Supplementary Material.

A custom three-column format convolved with a gamma hemodynamic response function and its temporal derivative was used to model the data. For the covert faces task, the EVs included “fear”, “angry”, and “happy” faces. Contrasts analysed included means for each condition, mean across all emotions and directional comparisons.

The main contrast of interest for the checkerboard task was flashes vs. baseline. For both task analyses motion parameters estimated by MCFLIRT (Motion Correction by FMRIB’s Linear Image Registration Tool) were added to the model as nuisance regressors. Absolute and relative motion values did not differ significantly between groups and no participant demonstrated significant movement (all included participants revealed absolute and relative motion <1.5 mm).

Registration to high-resolution structural images was carried out using FLIRT (FMRIB’s Linear Image Registration Tool) and further refined using FNIRT (FMRIB’s Non-Linear Image Registration Tool) nonlinear registration. Data were normalized to the Montreal Neurological Institute (MNI) template [32–35].

Higher level (group level) analysis was carried out using FSL’s tool for nonparametric permutation inference Randomise (5000 permutations) [36] to assess general effects of task-relevant contrasts on both groups, as well as test for group differences. Statistics were assessed using the threshold-free cluster enhancement method with family-wise error correction of 0.05 (or 0.95 threshold within randomise) [37]. The general linear model (GLM) included 2 groups: placebo and lamotrigine. Contrasts were defined as placebo greater than lamotrigine, lamotrigine greater than placebo, and the mean across both groups. To account for the possibility that differences in self-reported anxiety and side effects between the two groups could drive the neural difference, demeaned post-scan state anxiety scores (not pre-scan state anxiety scores due to missing values, no significant difference between pre- and post-scan state anxiety scores) and side effect ratings (VAS alertness, calmness, drowsiness, and dizziness), were added as regressors of no interest.

Significant brain areas were extracted for visualization using the fslmaths and cluster tools, with a threshold of 0.95 (based on the 1-p thresholding from randomise, described above). To further visualise results, individual parameter estimate (PE) values were extracted from their custom maps, using significant clusters as binary masks. In addition, left and right amygdala masks were created based on the Harvard-Oxford Atlas (thresholded at 50), and PE values were again extracted for visualisation. Activations are reported using MNI coordinates, and brain regions are reported based on the Harvard-Oxford Cortical and Subcortical Structural Atlas.

## Results

### Demographic and baseline clinical characteristics

Demographic and baseline clinical characteristics are presented in Table 1. Participants were recruited between the May 2017 and November 2018. The trial ended after the 36^th^ participant was enrolled. The final study sample consisted of 31 participants (lamotrigine *n* = 14, placebo *n* = 17) as 5 participants’ data were not able to be analysed due to significant artefacts caused by movement during the scan (*n* = 2, one from lamotrigine and placebo group each), incidental findings identified during pre-processing (*n* = 1 from lamotrigine group), ghosting artefacts (*n* = 1 from lamotrigine group), and a software error (*n* = 1 from lamotrigine group).

**Table 1:** Sociodemographic, clinical, and personality characteristics.

	*Placebo (n* = *17)*	*Lamotrigine (n* = *14)*
Gender		
Male	*n* = 11	*n* = 9
Female	*n* = 6	*n* = 5
Age	24.65 (4.3)	23.21 (4.4)
Verbal IQ (NART)	118.59 (5.4)	118.36 (3.9)
Beck Depression Inventory (BDI)		
Screening visit	1.82 (1.9)	3.36 (3.3)
Testing visit baseline	1.41 (1.9)	3.43 (2.6)
PANAS		
Positive (baseline)	34.29 (7.0)	32.14 (6.2)
Negative (baseline)	11 (1.1)	11.36 (1.4)
Positive (pre-scan)	30.38 (8.7)	26.93 (7.7)
Negative (pre-scan)	10.75 (1.1)	11.07 (1.6)
Positive (post-scan)	33.81 (8.3)	28.71 (7.9)
Negative (post-scan)	10.5 (1.1)	12.14 (5.8)
Spielberger State and Trait Anxiety Inventories		
Trait-Anxiety (screening visit)	30.00 (5.0)	34.36 (4.6)
State-Anxiety (baseline	25.06 (5.9)	30.0 (5.9)*
State-Anxiety (pre-scan)	26.94 (6.0)	31.29 (6.8)*
State-Anxiety (post-scan)	25.29 (5.4)	32.00 (9.6)*
Eyseneck Personality Questionnaires (EPQ)		
Neuroticism	4.81 (3.0)	7.71 (4.9)
Psychoticism	2.63 (2.5)	3.00 (1.8)
Extraversion	15.88 (3.8)	14.00 (4.9)
Lie/Social Desirability	8.81 (3.4)	9.50 (4.9)
Perceived lamotrigine treatment (%)		
Participant impression	11.76	64.29
Researcher impression	5.88	64.29

Values are means (SD).

Time points: baseline = prior to drug administration.

Pre-scan = 2.5 h post drug/placebo administration.

Post-scan = 3.5 h post drug/placebo administration.

*NART* National Adult Reading Test, *EPQ* Eysenck Personality Questionnaire, *BDI* Beck Depression Inventory, *PANAS* Positive and Negative Affective Schedule.

*p < 0.05.

The lamotrigine and placebo groups were well matched on sociodemographic, clinical, and personality parameters. Given the randomised nature of the study design, statistical analysis on the homogeneity of treatment groups at baseline was not carried out [38].

### Subjective ratings

There were no significant interaction effects between time and treatment on self-reported state anxiety and mood ratings as measured by the STAI and PANAS (all F’s < 0.75, p’s > 0.1, η2’s < 0.080) (Table 1). However, there was a significant difference between groups on state anxiety across all time points (F_(1,28)_ = 6.54, *p* = 0.016, η2 = 0.189), where the lamotrigine group had a significantly greater mean state anxiety score (M = 31.1, SD = 7.5) than the placebo group (M = 26.0, SD = 5.7).

There was a significant main effect of group on VAS ratings of calmness as well (F_(1,28)_ = 6.24, *p* = 0.019, η2 = 0.182) with the lamotrigine group (mean, SD = 66.3, 27.9) reporting feeling significantly less calm (e.g., more excited and tense) across all timepoints compared to the placebo group (mean, SD = 41.8, 29.9), even prior to drug treatment (Table 2).

**Table 2:** Reported side effects at baseline, pre-scan, and post-scan.

	Baseline				Pre-scan				Post-scan			
	Placebo Mean ± SD		Lamotrigine Mean ± SD		Placebo Mean ± SD		Lamotrigine Mean ± SD		Placebo Mean ± SD		Lamotrigine Mean ± SD	
VAS												
Alertness	207.1	124.5	242.6	116.7	242.4	59.6	419.9*	31.3	248.8	27.3	343.7	33.0
Satisfaction	88.9	127.2	101.1	156.4	84.4	62.8	125.0	38.5	87.3	29.3	119.2	39.9
Calmness	42.5	147.8	65.1*	150.6	41.3	65.7	58.9*	79.5	41.5	33.0	75.0*	44.9
Side effects												
Nausea	0	0	0	0	0.06	0.24	0.14	0.36	0	0	0.14	0.36
Dry mouth	0.12	0.33	0.07	0.27	0.12	0.33	0.29	0.61	0.12	0.33	0.21	0.43
Agitation	0	0	0.07	0.27	0	0	0	0	0	0	0.14	0.53
Aggression	0	0	0	0	0	0	0	0	0	0	0.07	0.27
Headache	0	0	0.14	0.36	0.12	0.33	0.29	0.61	0	0	0.29	0.47
Drowsiness	0.12	0.33	0.14	0.36	0.24	0.44	0.71*	0.73	0.24	0.56	0.79*	0.80
Dizziness	0	0	0.07	0.27	0	0	0.71*	0.91	0.06	0.24	0.57*	0.76
Tremor	0	0	0	0	0.06	0.24	0.14	0.36	0	0	0.07	0.27
Back/joint pain	0	0	0.07	0.27	0	0	0	0	0	0	0	0
Vision	0	0	0	0	0	0	0.07	0.27	0	0	0	0
Rash	0	0	0	0	0	0	0.14	0.53	0	0	0.07	0.27

*p < 0.05.

There was also significant group by timepoint interaction for VAS ratings of alertness (F_(2,56)_ = 6.67, *p* = 0.003, η2 = 0.192) (see also Table 2). Post-hoc comparisons showed that the lamotrigine group (mean = 420, SD = 156) reported feeling less alert (e.g., more drowsy, clumsy, and lethargic) than the placebo group (mean = 242, SD = 127) pre-scan (after drug administration) (*p* = 0.002). There were no significant group differences at baseline or post-scan (p’s > 0.093). The groups did not differ on VAS ratings of satisfaction (F’s < 2.27, p’s > 0.143, η2’s < 0.139) (Table 2).

There was a significant time by condition interaction for side effects (F_(2,58)_ = 5.95, *p* = 0.004, η2 = 0.170) with the lamotrigine group presenting significantly more side effects than the placebo group after treatment (both pre-scan (*p* = 0.004) and post-scan (*p* = 0.007)) but not at baseline (*p* = 0.277) (Table 2). Specifically, participants in the lamotrigine group reported higher scores on drowsiness (pre-scan: *p* = 0.031; post-scan: *p* = 0.033) and dizziness (pre-scan: *p* = 0.003; post-scan: *p* = 0.013) as reflected in a significant condition by time by side effect interaction (F_(20,580)_ = 1.97, *p* = 0.007, η2 = 0.064).

### Effectiveness of blinding

All participants and the researcher conducting the study visit were asked to guess whether the participant had been administered lamotrigine or a placebo. The impression of group was not completed for one subject who was in the placebo group. Findings reveal that both participants (X2 _(1)_ = 8.62, *p* = 0.003) and the researcher (X2 (1) = 11.32, *p* = 0.001) were able to correctly identify the subject’s condition significantly more often than not (Table 1).

### Behavioural task performance

Accuracy in correctly identifying gender during the fMRI faces task was overall high ( > 95%, Table 3), confirming that participants were engaged in the task. However, there was also a significant interaction between emotion and treatment (F_(2,58)_ = 4.924, *p* = 0.011, η2 = 0.145). Post-hoc tests revealed that this effect was driven by fearful faces, with the lamotrigine group being more accurate than the placebo group (*p* = 0.045) at classifying gender for faces showing this emotion, but not anger (*p* = 0.441) nor happiness (*p* = 0.281). In addition, participants in the placebo group performed worst for fearful faces overall, reflected in trend significant differences between fear vs happy (*p* = 0.081) and fear vs anger (*p* = 0.052), but not happy vs anger *p* = 0.851). There was no significant difference between the lamotrigine-treated and placebo-treated groups in reaction time (F_(1,29)_ = 0.06, *p* = 0.806, η2 = 0.002), nor an interaction between emotion and treatment (F_(2,58)_ = 1.33, *p* = 0.272, η2 = 0.044).

**Table 3:** Accuracy and reaction times on the faces task.

	Placebo Mean ± SD	Lamotrigine Mean ± SD	Total Mean ± SD
Accuracy			
Fear	95.29 (4.3)	97.86 (1.7)*	96.45 (3.6)
Happy	97.06 (3.9)	95.71 (2.7)	96.45 (3.4)
Anger	97.21 (2.9)	96.43 (2.5)	96.85 (2.7)
Reaction times			
Fear	497.13 (84.5)	498.78 (85.4)	497.87 (83.5)
Happy	496.03 (94.9)	483.27 (85.1)	490.27 (89.3)
Anger	494.55 (89.7)	482.83 (76.8)	489.26 (82.9)

*p < 0.05.

### fMRI results

#### Main effect of task—faces task

To determine if the task engaged brain regions previously associated with emotional and facial stimuli, neural activation in response to fear vs. baseline, happy vs. baseline, anger vs. baseline and mean faces vs. baseline was compared across groups. A whole-brain analysis revealed significant activation in response to emotional stimuli vs. baseline across groups in a network of areas (Supplementary Fig. 1). Significant activity was observed in clusters that include the occipital fusiform gyrus, bilateral amygdala, angular gyrus and ACC. These results are consistent with previous reports using the same task, suggesting that the task was successful in probing emotional processing [26, 30, 39].

#### Effect of lamotrigine administration

**Fig. 3:**
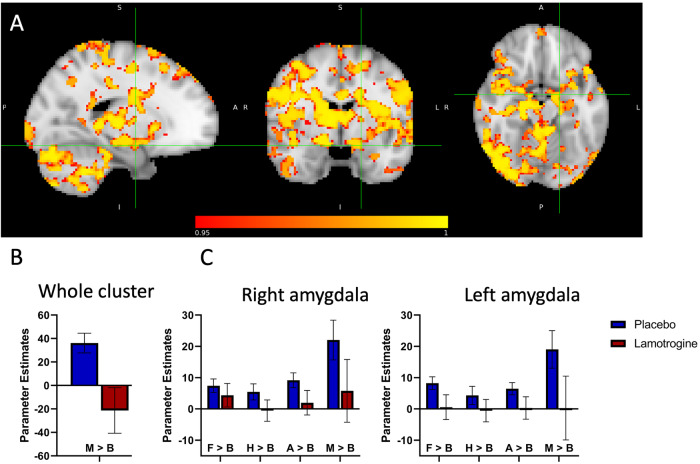
fMRI Faces Task results. **A** Sagittal, coronal, and axial images depicting significantly reduced BOLD activation (deactivation) in the lamotrigine group relative to placebo in response to mean of all faces versus baseline, in a whole range of areas including bilateral amygdala, insula and ACC. Cursor in the left amygdala MNI coordinates: x = −18, y = −4, z = −16. Results are shown TFCE-corrected with a family-wise error cluster significance of 1 – p > 0.95. **B** Parameter estimates extracted from the whole cluster, and **C** left and right amygdala anatomical mask thesholded at 50. Error bars represent the standard error of the mean.

A whole-brain analysis revealed a range of areas with reduced BOLD activation in the lamotrigine group relative to placebo, as a main effect of group in response to the mean of all faces versus baseline (78236 voxels, peak voxel location: x = 12, y = −12, z = 16 right thalamus, t-max = 5.53, *p* = 0.001)). These brain areas include bilateral amygdala, hippocampus, ACC, insula, superior temporal gyrus, anterior PFC, frontal medial cortex, paracingulate gyrus, nucleus accumbens, posterior cingulate cortex (PCC), precuneous cortex and pre-and post-central gyrus (Fig. 3 and Supplementary Table 1). Similar results were found for each individual emotion (vs. baseline) (Supplementary Table 1). No group differences were seen for the contrasts comparing the different emotions with each other (i.e., a group x emotion interaction).

#### Visual checkerboard

In the checkerboard task, visual stimulation was associated with a number of significant activation clusters, across groups. These included the bilateral occipital cortex, superior parietal lobule towards angular gyrus, supramarginal gyrus, right precentral gyrus, right middle frontal gyrus and right inferior frontal gyrus (Supplementary Fig. 2). No effects of lamotrigine were found suggesting that the observed effects on emotional processing did not reflect global haemodynamic changes.

#### Sensitivity analysis

Adding demeaned subjective ratings of alertness, calmness, dizziness, or drowsiness as a nuisance regressor together with state anxiety in the fMRI analysis resulted in similar results. When correcting just for alertness or drowsiness the main effect of group (mean all emotions > baseline) remains in amygdala and ACC (Supplementary Fig. 3).

When adding state anxiety as a covariate (as per imaging analysis) and/or drowsiness, the accuracy and reaction time results of the gender identification task remain similar, accuracy: F’s > 4.21, p’s < 0.020; reaction time: F’s < 0.91, p’s > 0.410.

## Discussion

The main aim of the current study was to investigate the effects of a single dose of lamotrigine on subjective mood and neural measures of emotional processing in healthy volunteers. Participants in the lamotrigine group were more accurate at identifying the gender of fearful faces compared to the placebo group. In addition to this behavioural effect, we saw a reduction in BOLD activity in the lamotrigine group relative to placebo in response to fearful, happy, and angry faces (versus baseline) in a network of regions associated with emotional processing, including the amygdala, insula and ACC. Therefore, not in line with our hypothesis, lamotrigine had valence-independent rather than emotion-specific effects on neural measures of emotional processing. There were no effects of lamotrigine in response to visual stimulation, supporting the conclusion that the effects on emotional processing were not driven by global drug-related modulation of the BOLD signal. Undesired side effects related to lamotrigine included reduced alertness and increased drowsiness. However, neural results were not affected by adding subjective ratings as nuisance regressors. Together these findings indicate that lamotrigine has broad ranging effects on neural response to emotional stimuli. This would suggest an effect of lamotrigine that is not specific to negative emotional stimuli, at least at a neural level, which may be relevant for models of mood stabilising action to consider.

The amygdala and ACC play an important role in emotional processing, and are postulated as a key site of traditional antidepressant action [19]. Specifically, acute doses of conventional antidepressants have been shown to reproducibly and significantly reduce amygdala response to fearful faces and/or increase activation to happy faces in this region [20, 26, 40] perhaps reflecting early normalisation of negative affective bias in depression. These changes in emotional processing have been associated with later improvements in mood, suggesting that they represent a critical pathway through which antidepressants exert their effects [23, 25]. In our current study lamotrigine reduced BOLD activity in the amygdala and ACC in response to both positive and negative faces. The amygdala plays a key role in detecting salient information in the environment [41, 42]. It is therefore possible that this effect of reducing activity in the amygdala in response to both positive and negative valences could contribute to the mood-stabilising effect of lamotrigine, given that patients with BD suffer from mood instability and their interpretation of positive and negative events can oscillate greatly between mood episodes. This hypothesis remains speculative however and further research is needed to further explore this.

Moreover, group differences between lamotrigine and placebo were also seen in other areas, including areas related to motor control and movement, attention, and cognition. In addition, activity in areas related to emotional processing and attention other than the amygdala and ACC, such as insula, anterior PFC, PCC, precuneous, supramarginal gyrus and paracingulate gyrus, was also significantly reduced in the lamotrigine group. These findings indicate that lamotrigine may reduce brain activity in several areas related to emotional processing in a different way than other studies using SSRI antidepressants have reported. As lamotrigine regulates glutaminergic release by inhibiting voltage-gated channels associated with glutamate, it is perhaps not surprising that widespread changes in activation patterns are seen. Interestingly the areas identified overlap with regions that form the default mode network (DMN), the salience network (SAN) and the affective network (AN). These resting state networks integrate cognitive control, affective and reward-systems of the brain and have been implied in the pathophysiology of bipolar disorder [43–45]. The glutamatergic system also plays an important role in regulating these networks [46–50]. For example, it has been shown that high glutamate concentration in the PCC and precuneous area is associated with reduced deactivation of the DMN [51].

Behaviourally lamotrigine improved the performance on a simple gender discrimination task for fearful faces only. Typically healthy volunteers are less accurate and slower at identifying the gender of fearful faces than that of happy faces in this task (e.g., [39]), suggested to reflect a distraction effect from the threat relevant content of the fearful faces interfering with the unrelated decision regarding facial gender. The improved accuracy in classifying the fearful faces in those receiving lamotrigine could represent reduced threat distraction by the fearful face content, even in the absence of differences in neural response to fear vs happy faces. Difficulties in reducing activation within the DMN has been linked to increased rumination and impaired control of action [45, 52, 53]. Thus, the pattern of decreased DMN activation and reduced fear distraction following lamotrigine appears consistent and highlights a potential mechanism of action. Future resting state studies may clarify our findings further.

To our knowledge this is the first study that has investigated the neural effects of lamotrigine in healthy volunteers. As part of the same trial as this current study, we also investigated the effects of lamotrigine on behavioural measures of emotional processing in different cognitive domains, using the Emotional Test Battery (ETB) [29]. The ETB is a validated tool also proven to be sensitive to the early effects of antidepressant medication [17, 18]. There were no effects on emotional face recognition, in contrast to the effects seen here in neural response to face stimuli. Such a difference may occur because of increased sensitivity of fMRI measures, which are closer to mechanistic sites of action, compared to behavioural readouts. However, the behavioural results from the ETB also suggested a decrease in negative vs positive memory recall following lamotrigine [54]. The effects of lamotrigine on emotional processing are therefore likely complex and may reflect dissociable effects on neural circuity underpinning these different perceptual vs memory functions. Alternatively, it is possible that reducing the impact of emotional salient stimuli (shown here with reduced neural reactivity) could have the behavioural consequence of releasing more memory capacity to recall the relatively more positive items i.e., reducing the priority often given to negative stimuli.

The effect of lamotrigine on brain activity has been somewhat more widely explored in the context of emotional and cognitive tasks in patients with bipolar disorder. In terms of resting state functional connectivity (rsFC) a recent study by [55] suggested that preserved rsFC between the frontoparietal network (FPN) and the dorsal attention network (DAN) (the networks involved in cognitive control), and the hub of the posterior DMN (the precuneus), was critical for good response to lamotrigine as an add-on treatment in patients with bipolar depression. With regards to task-based fMRI, some studies have suggested a link between symptomatic improvement and normalization and therefore increased BOLD activity in a number of brain regions, including the PFC and ACC. However, sample sizes were small and most of these studies included children and adolescents [56–58]. Turning to other pharmacological agents that influence downstream levels of glutamate, results have been mixed. For example, ebselen is a bioavailable antioxidant shown to lower glutamate levels in the ACC in healthy volunteers [59, 60]. Ebselen has been found to differentially influence the recognition of positive vs. negative facial expressions in the Facial Expression Recognition Task (FERT), a behavioural measure of emotional processing in one study [61], but not in another [62]. Experimental medicine studies with neural outcome measures of emotional processing are currently underway [60]. Ketamine on the other hand is a non-competitive N-methyl-D-aspartate (NMDA) receptor antagonist causing increased presynaptic glutamate release and extracellular glutamate concentrations. Similar to the results from the current study, ketamine has been shown to reduce neural reactivity in the amygdala after emotional stimulation with both positive and negative pictures [63, 64]. However, ketamine and lamotrigine would be expected to have a different profile of effect on the glutamate system suggesting that the relationship between emotional processing and glutamate is likely to be complex.

Several factors that may have influenced the current study must be taken into consideration when interpreting results. This includes significant differences between groups in self-report clinical data, as well as significant differences in behavioural task performance between groups, and unsuccessful participant and researcher blinding. The lamotrigine and placebo groups in our study differed significantly in several important assessments, including subjective state anxiety and side-effect profile. This may have served as a confounding factor that interacted with emotional processing and neural response. We accounted for this by adding these subjective measures as nuisance regressors. In addition, further unknown between-group differences could also have adversely impacted the results and it is therefore not feasible to attribute group differences to solely the effect of lamotrigine. It could be hypothesized that the lamotrigine group’s significantly reduced neural response to emotional stimuli resulted from the participants not processing the stimuli as much. This could be due to the lamotrigine group being significantly more anxious than the placebo group, or the lamotrigine group experiencing significantly greater negative side effects, including greater drowsiness and reduced alertness. It would be possible that lamotrigine group’s anxiety and reduced arousal caused them to avoid the emotional stimuli compared to the placebo group and therefore explain the lamotrigine group’s reduced neural response to presented emotional faces compared to baseline. However, the lamotrigine group was significantly better at identifying gender of fearful faces which argues against this interpretation. A within-subjects design would have been able to overcome some of the difficulties with group matching via randomisation, however a parallel-group design was utilised to avoid the possibility of practice effects, and habituation to the emotional stimuli used in this task. Finally, the relatively small sample size (*n* = 31) may have impacted the power of the study to detect broader effects of lamotrigine on emotional pressing, in addition to type 2 errors. The generalisability of the current results to clinical populations is limited. Using healthy participants for early mechanistic work in humans has a number of advantages; in particular, it allows the characterisation of neurocognitive effects without confounding due to symptom change. However, prospective conclusions about the potential effect of lamotrigine on emotional processing and their relevance for the treatment of bipolar disorder remain to be validated in future work that uses a well-matched, high-powered sample of patients across a broader age range.

## Conclusion

A single dose of lamotrigine significantly reduced activation in a range of brain areas important for emotional processing, including bilateral amygdala and ACC, in response to both positive and negative emotions. Therefore, lamotrigine has an effect that seems to be valence-independent rather than valence-specific. Together, while also considering the limitations of the current study, this suggests lamotrigine has a broad-acting mechanism of action that is not specific to negative emotional stimuli. This data is important as it suggests a mechanism of action underlying lamotrigine that may be relevant to its mood stabilisation effects. Future research in a larger sample is needed to further clarify the neural mechanisms underlying lamotrigine administration, their relevance for the treatment of bipolar disorder and the translation to unipolar depression.

### Supplementary information

Supplementary material

## Data Availability

Anonymised subjective and behavioural data, as well as normalised MRI data (such that unique brain structure is not shared) that support the findings of this study, are available from the corresponding author MM, upon reasonable request. The data are not publicly available due to restrictions on consent given by participants.
