# Supplementary material for: Acute neural effects of the mood stabiliser lamotrigine on emotional processing in healthy volunteers: a randomised control trial
Source: Transl Psychiatry. 2024 May 27;14:211. doi: 10.1038/s41398-024-02944-6 (PMC11130123; doi:10.1038/s41398-024-02944-6)
Supplement: Supplementary file 1 — Supplementary material [file 41398_2024_2944_MOESM1_ESM.docx]

**Acute neural effects of the mood stabiliser lamotrigine on emotional processing in healthy volunteers – a randomised control trial**

Methods:

Participants were fluent in English and healthy. Participants were excluded if any of the following applied:

- Current usage of psychoactive medication;
- Current usage of the contraceptive pill;
- Any past or current psychiatric disorder (assessed by SCID-5);
- Significant medical condition;
- Current pregnancy or breastfeeding;
- Current or history of drug or alcohol dependency;
- Participation in a psychological or medical study involving the use of medication within the last 3 months;
- Previous participation in a study using the same, or similar, emotional processing tasks;
- Smoker > 5 cigarettes per day;
- Typically drinks > 6 caffeinated drinks per day;
- Takes folic acid supplements;
- History of recurrent allergies and rashes;
- Contraindication to MRI (e.g., metallic implants);

**Neuroimaging protocol:**

The neuroimaging protocol comprised functional and structural sequences as follows. Structural scans were acquired via T1-weighted MR images (TR=1900ms, TE=3.97ms, flip angle=8°, field of view =192mm, voxel dimension = 1mm3 voxels, acquisition time = 5min 31 seconds). Functional imaging consisted of 72 T2-weighted echoplanar imaging (EPI) slices (TR=1200ms, TE=30ms, flip angle=65°, slice thickness=2mm, multiband accelerator factor 4, PAT GRAPPA factor 2, resolution = 2mm isotropic. Images were distortion corrected by an acquired fieldmap (echos at 4.92 and 7.38ms, TR=482ms, flip angle=46°).

**fMRI task designs**

The faces task (also called gender discrimination task) was designed to probe emotional processing and has proved sensitive to the acute effects of antidepressants on neural processing [1,2]. The task was a block design presenting colour photographs of faces expressing three emotions (fear, happy, anger) taken from the NimStim database (Tottenham et al., 2009). In each trial, an emotional face was presented for 100ms followed by a fixation cross for 2900ms. The task contained four blocks of each valence, each block lasting 30 seconds, interspersed with fixation cross blocks lasting 30 seconds each. Participants were asked to respond by indicating the gender (male or female) of each face as quickly and accurately as possible via button press. Reaction time (total time between face stimuli presentation and gender classification response) and accuracy (number of faces correctly identified as male/female divided by total number of faces) were measured. This task has proved sensitive to the acute effects of antidepressants on neural processing.

Following the faces task, a checkerboard visual paradigm was presented. This assessed the effect of lamotrigine on the blood oxygen level-dependent (BOLD) signal in the primary visual cortex, to control for a possible confounding effect of global drug-related modulation of BOLD signal. The paradigm was a passive visual task in which participants viewed flashing blocks of two alternating and opposite configurations of black and white squares that switched at a frequency of 8Hz. These blocks lasted for 15 seconds and were separated by 15 seconds of fixation cross for a total of 20 cycles. The flashing checkerboard blocks were introduced from a homogeneous grey background using a gradient to avoid startling participants.

**fMRI data analysis - pre-processing:**

fMRI data were pre-processed and analysed using FEAT (FMRI Expert Analysis Tool). The following pre-statistics processing was applied to reduce unwanted variability in the data and improve validity of statistical analyses: motion correction using FMRIB’s Linear Image Registration Tool (MCFLIRT) [3,4], non-brain removal using BET [5], spatial smoothing using a Gaussian kernel of 5mm full-width-half maximum, grand-mean intensity normalization of the entire 4D dataset by a single multiplicative factor, high pass temporal filtering (Gaussian-weighted least-squares straight line fitting, sigma=45.0s), and B0 unwarping using fieldmap phase and magnitude images for distortion correction [6,7].

**Figure S1:** Activation of brain areas during the fMRI emotional faces task. The sagittal, coronal, and axial images depict neural activation in response to (A) fearful faces versus baseline (red), (B) happy faces versus baseline (green), (C) angry faces versus baseline (blue) and (D) mean of the three valences versus baseline, across both treatment groups. Cursor in left amygdala MNI: x = -19, x = -5, y = -14. Results are shown TFCE-corrected with a family-wise error cluster significance level of 1 – p > 0.95.

**
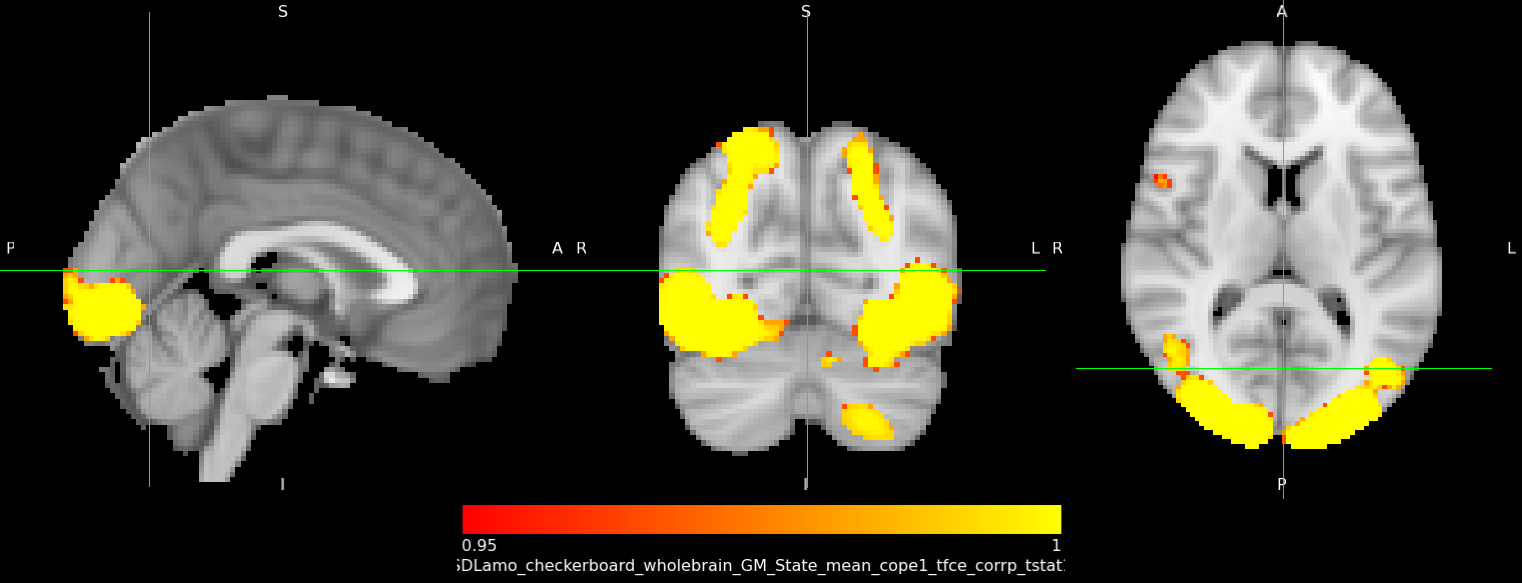
Figure S2:** Main effect of task visual stimulation checkerboard task. Sagittal, coronal, and axial images depicting neural activation in response to flashes versus baseline, across groups. Cursor MNI coordinates: x = 0, y = -70, z = 10. Results are shown TFCE-corrected with a family-wise error cluster significance level of 1 – p > 0.95.

**Figure S3:** Sagittal, coronal, and axial images depicting significantly reduced BOLD activation in the lamotrigine group relative to placebo in response to mean of all faces versus baseline, in a whole range of areas including amygdala.

1. Corrected for subjective ratings of alertness (VAS)
2. Corrected for subjective measures of calmness (VAS)
3. Corrected for subjective ratings of drowsiness (side effects questionnaire)

Cursor in the left amygdala MNI coordinates: x = -18, y = -4, z = -16. Results are shown TFCE-corrected with a family-wise error cluster significance of 1 – p > 0.95.

Table S1 - Areas of significant decrease in BOLD response for the lamotrigine group compared to placebo during emotional processing (whole brain analysis).

|  | **Brain area** | **Cluster size (voxels)** | | **MNI max**  **(x, y, z)** | | | ***t* score** | ***p*-value** |
| --- | --- | --- | --- | --- | --- | --- | --- | --- |
| Group differences, TFCE-corrected FWE cluster significance level of p<0.05  Corrected for state anxiety ratings. | | | | | | | | |
| **Placebo > lamotrigine** | | |  |  | | |  |  |
| Mean all emotions > baseline | | |  |  | | |  |  |
| Cluster 1 | Including bilateral thalamus, **amygdala,** hippocampus, **insula**, nucleus accumbens, **ACC**, PCC, precuneous, frontal pole, frontal medial cortex, paracingulate gyrus, pre- and post-central gyrus, superior parietal lobule, supramarginal gyrus, angular gyrus | | 78236 | 12 | -12 | 16 | 5.53 | 0.001 |
| Fear > baseline | | |  |  |  |  |  |  |
| Cluster 1 | Including bilateral thalamus, **amygdala,** hippocampus, **insula**, **ACC**, PCC, precuneous, frontal pole, paracingulate gyrus, pre- and post-central gyrus, superior parietal lobule, supramarginal gyrus, angular gyrus | | 65400 | -12 | -18 | 0 | 6.51 | <0.001 |
| Happy > baseline | | |  |  |  |  |  |  |
| Cluster 1 | Including bilateral cerebellum, **amygdala**, hippocampus, **insula**, **ACC**, sgACC, PCC, precuneous, frontal pole, paracingulate gyrus, pre- and post-central gyrus, superior parietal lobule, supramarginal gyrus, angular gyrus | | 63892 | -36 | -62 | -36 | 5.18 | 0.001 |
| Anger > baseline | | | | | | | | |
| Cluster 1 | Bilateral cerebellum | | 2929 | 22 | -46 | -40 | 6.16 | 0.024 |
| Cluster 2 | Bilateral **ACC**, paracingulate gyrus, superior frontal gyrus | | 477 | -8 | 6 | 40 | 3.95 | 0.041 |
| Cluster 3 | Right post-central gyrus, pre-central gyrus, middle frontal gyrus | | 360 | 44 | -10 | 30 | 4.81 | 0.034 |
| Cluster 4 | Right frontal orbital cortex | | 84 | 30 | 18 | -26 | 5.34 | 0.033 |
| Cluster 5 | Right **insula** | | 70 | 38 | 20 | -8 | 4.32 | 0.043 |
| Cluster 6 | Cerebellum | | 69 | -10 | -82 | -36 | 4.17 | 0.044 |
| Cluster 7 | Right supramarginal gyrus | | 57 | 46 | -38 | 40 | 4.22 | 0.045 |
| Cluster 8 | Bilateral superior frontal gyrus | | 51 | 2 | 36 | 46 | 3.65 | 0.046 |
| Cluster 9 | Right Cerebellum | | 48 | 42 | -60 | -54 | 3.82 | 0.045 |
| Cluster 10 | Right paracingulate gyrus extending in superior frontal gyrus | | 46 | 6 | 46 | 28 | 4.32 | 0.044 |
| Cluster 11 | Left caudate | | 35 | -10 | 8 | 2 | 4.06 | 0.046 |
| Cluster 12 | Right lateral occipital cortex | | 22 | 46 | -80 | -12 | 4.45 | 0.046 |
| Not significant  p < 0.10 | Right **amygdala** | | 60 | 22 | 0 | -14 | 3.60 | 0.066 |
| MNI coordinates (*x, y, z*) refer to the peak of activation within each cluster | | | | | | | | |

**References**

1. Capitão LP, Chapman R, Murphy SE, Harvey C-J, James A, Cowen PJ, et al. A single dose of fluoxetine reduces neural limbic responses to anger in depressed adolescents. Transl Psychiatry. 2019;9:1–9.

2. Martens MAG, Kaltenboeck A, Halahakoon DC, Browning M, Cowen PJ, Harmer CJ. An Experimental Medicine Investigation of the Effects of Subacute Pramipexole Treatment on Emotional Information Processing in Healthy Volunteers. Pharmaceuticals. 2021;14:800.

3. Jenkinson M, Bannister PR, Brady M, Smith SAC. Improved Optimization for the Robust and Accurate Linear Registration and Motion Correction of Brain Images. NeuroImage. 2002;17:825–841.

4. Jenkinson M, Smith S. A global optimisation method for robust affine registration of brain images. Medical Image Analysis. 2001;5:143–156.

5. Smith SM. Fast robust automated brain extraction. Human Brain Mapping. 2002;17:143–155.

6. Jenkinson M. Fast, automated, N-dimensional phase-unwrapping algorithm. Magnetic Resonance in Medicine. 2003;49:193–197.

7. Jenkinson M. Improving the registration of B0-disorted EPI images using calculated cost function weights. Tenth International Conference on functional mapping of the human brain, 2004.
